# Effects of recombinant human growth hormone treatment on growth, body composition, and safety in infants or toddlers with Prader-Willi syndrome: a randomized, active-controlled trial

**DOI:** 10.1186/s13023-019-1195-1

**Published:** 2019-09-11

**Authors:** Aram Yang, Jin-Ho Choi, Young Bae Sohn, Yunae Eom, Jiyoon Lee, Han-Wook Yoo, Dong-Kyu Jin

**Affiliations:** 1 Department of Pediatrics, Kangbuk Samsung Hospital, Sungkyunkwan University School of Medicine, Seoul, Republic of Korea; 2 Department of Pediatrics, Asan Medical Center Children’s Hospital, University of Ulsan College of Medicine, 88 Olympic-ro 43-gil, Songpa-gu, Seoul, 05505 Republic of Korea; 3 Department of Medical Genetics, Ajou University Hospital, Ajou University School of Medicine, Suwon, Republic of Korea; 4 Life Sciences, LG Chem, Ltd, Seoul, Republic of Korea; 5 Department of Pediatrics, Samsung Medical Center, Sungkyunkwan University School of Medicine, 81 Irwon-ro, Gangnam-gu, Seoul, 06351 Republic of Korea

**Keywords:** Prader-Willi syndrome, Growth hormone therapy, Body composition, Psychomotor development, Infants and toddlers

## Abstract

**Background:**

Prader-Willi syndrome (PWS) is a rare complex genetic disorder and is characterized by short stature, muscular hypotonia, abnormal body composition, psychomotor retardation, and hyperphagia. Recombinant human growth hormone (rhGH) treatment improves the symptoms in children with PWS, and early treatment results in more favorable outcomes. However, systematic studies in infants and toddlers under 2 years of age are lacking. This multicenter, randomized, active-controlled, parallel-group, open-label, Phase III study aimed to evaluate the safety of rhGH (Eutropin, LG Chem, Ltd.) and its efficacy on growth, body composition, and motor and cognitive development in infants and toddlers with PWS compared with a comparator treatment (Genotropin, Pfizer, Inc.). Eligible Korean infants or toddlers with PWS were randomly assigned to receive Eutropin or comparator (both 0.24 mg/kg/week, 6 times/week) for 1 year. Height standard deviation score (SDS), body composition, and motor and cognitive development were measured.

**Results:**

Thirty-four subjects (less than 24 months old) were randomized into either the Eutropin (*N* = 17) group or the comparator (*N* = 17) group. After 52 weeks of rhGH treatment, height SDS and lean body mass increased significantly from baseline in both groups: the mean height SDS change (SD) was 0.75 (0.59) in the Eutropin group and 0.95 (0.66) in the comparator group, and the mean lean body mass change (SD) was 2377.79 (536.25) g in the Eutropin group and 2607.10 (641.36) g in the comparator group. In addition, percent body fat decreased significantly: the mean (SD) change from baseline was − 8.12% (9.86%) in the Eutropin group and − 7.48% (10.26%) in the comparator group. Motor and cognitive developments were also improved in both groups after the 1-year treatment. The incidence of adverse events was similar between the groups.

**Conclusions:**

rhGH treatment for 52 weeks in infants and toddlers with PWS improved growth, body composition, and motor and cognitive development, and efficacy and safety outcomes of Eutropin were comparable to those of Genotropin. Hence, Eutropin is expected to provide safe and clinically meaningful improvements in pediatric patients with PWS**.**

**Trial registration:**

The study was registered at ClinicalTrials.gov (identifier: NCT02204163) on July 30, 2014.

URL: https://clinicaltrials.gov/ct2/show/NCT02204163?term=NCT02204163&rank=1

## Background

Prader-Willi syndrome (PWS), a rare complex genetic disorder arising from the loss of expression of genes in the paternally inherited chromosome 15q11-q13, can be caused by a deletion, a uniparental disomy, or an imprinting center defect [1–3]. PWS is characterized by muscular hypotonia, failure to thrive in infancy, short stature, psychomotor retardation, and hyperphagia resulting in severe obesity, as well as hypothalamic dysfunction, which may become apparent in later childhood [4–6]. Patients with PWS also have a peculiar body composition with a high body fat mass percentage and a low lean body mass (LBM). This has been observed even in underweight infants with PWS [7, 8].

Clinical symptoms in children with PWS—including decreased energy expenditure, abnormal body composition, short stature, delayed skeletal maturation, and lack of growth hormone (GH) secretion—were more similar to the symptoms observed in GH deficiency than to those of non-syndromic obesity [9–11]. Therefore, recombinant human GH (rhGH) treatment for improvement of the symptoms in children with PWS has been available and regularized since rhGH preparations were approved for use in children with PWS by the US FDA in 2000 and the EMA in 2001 [11].

GH treatment (GHT) in PWS has been shown to improve growth and body composition, causing a decrease in body fat and an increase in LBM. It has also been shown to improve motor and cognitive development [5, 12–23]. Therefore, treatment with rhGH has been applied to children with PWS along with diet adjustments, exercise prescription, and behavioral therapy [11].

Delays in the developmental milestones of children with PWS appear at an early age, and early rhGH treatment has been shown to improve mental and motor development, and adaptive functioning in young children [20, 21, 24, 25]. Lo et al. [25] and Dykens et al. [26] suggested the concept of “earlier is better”; receiving GHT before 12 months of age results in much improved cognitive function reflected in higher IQ scores. In line with the results of this investigation, several recent studies emphasize the importance of early GHT in order to yield more favorable outcomes. Hence, treatment should be initiated at a very young age, in agreement with the recent trend toward younger ages for starting treatment [14, 22, 23]. Based on this, most of the patients with PWS in Korea are treated with rhGH from a very young age, shortly after they are diagnosed with PWS. However, there is a lack of relevant data, and to our knowledge, no systematic research has been conducted on the growth, body composition, cognition, and motor functions of infants and toddlers under 2 years of age.

In accordance with the need for rhGH preparations in children with PWS, this clinical study was aimed at confirming that Eutropin (LG Chem, Ltd., Seoul, Republic of Korea) — a rhGH — improves body composition as well as height and helps with the motor and cognitive developments. This was done by assessing the efficacy and safety of Eutropin compared to Genotropin (Pfizer Inc., New York, USA) in children with PWS.

## Methods

### Patients

The following patients were included in this study: 1) prepubertal pediatric patients with PWS confirmed using methylation polymerase chain reaction (PCR) genetic testing; 2) pediatric patients naïve to rhGH treatments or previously treated with rhGH for less than 6 months (the last administration at least 6 months prior to screening); 3) pediatric patients without other causes for growth retardation except for PWS; 4) pediatric patients who were not being administered any drug that may have an effect on the secretion and actions of GH (estrogen, androgen, anabolic steroids, corticosteroids, gonadotropin-releasing hormone, analogs, thyroxine, aromatase inhibitors, etc.), anticonvulsants, or cyclosporin at screening, and who had not been administered any of these drugs within 6 months prior to screening. The complete list of inclusion and exclusion criteria are available in Additional file 1: Table S1.

### Design

This study was a multicenter, randomized, active-controlled, parallel-group, open-label, Phase III study conducted at 3 centers in the Republic of Korea from October 2014 to December 2017. The study was conducted in compliance with the ethical guidelines of the Declaration of Helsinki and Good Clinical Practices, and was approved by the institutional review board of each study site. Written informed consent was obtained from the legally authorized representatives of the patients, since all participants were under 2 years of age and were unable to read or understand writing. The study was registered at ClinicalTrials.gov (NCT02204163).

The study consisted of three periods: (1) a screening period, (2) randomization followed by a treatment period of 52 weeks, and (3) a follow-up period of 4 weeks. After screening, the eligible subjects were randomly assigned to two groups by the investigators (1:1 ratio) and received either the rhGH treatment (Eutropin) or the comparator rhGH treatment (Genotropin) [24] according to a random sequence. The random sequence was generated by a statistician using the stratified block randomization method, and scratch-off labels were used to implement the random sequence. Subsequently, subjects received the assigned treatment for 52 weeks. The investigational product was administered subcutaneously 6 times a week at bedtime by the caregivers who were trained in safe injection practices. The dose of the investigational product was allowed to be gradually increased up to 0.24 mg/kg/week starting from 0.084 mg/kg/week at the discretion of the investigator in consideration of the safety of the subject [27]. After the treatment period of 52 weeks, subjects were followed up at week 56 for safety monitoring.

### Measurement methods

Height (cm), weight (kg), and head circumference (cm) were measured at baseline and at weeks 16, 28, and 52. Height was obtained using a calibrated infantometer. Mean height and weight were expressed as standard deviation scores (SDS) for age and sex, according to a Korean reference [27]. Percent body fat (%), LBM (g), and bone mineral density (g/cm) were measured at baseline and at week 52 using a dual-energy X-ray absorptiometry (DEXA) following a standard procedure at each study site. Standard bone age (BA) was determined from the X-ray scans of the left hand or knee at baseline and at week 52 using the method of Greulich and Pyle [28].

Motor and cognitive developments were assessed at baseline and at weeks 28 and 52 using the Bayley scales of infant development (BSID-II [29]/Korean BSID-II). In this study, all assessments of motor and cognitive development were conducted by qualified independent blinded evaluators. Blood samples were also collected at baseline and at weeks 28 and 52 for assessment of serum insulin-like growth factor I (IGF-I) and IGF-binding protein 3 (IGFBP-3). IGF-I and IGFBP-3 levels were measured at a central laboratory using a validated electrochemiluminescence immunoassay (ECLIA) method, and these were transformed into SDS for age, according to laboratory reference values.

The primary endpoints were changes in height SDS, LBM, and percent body fat at week 52 from baseline. The secondary endpoints were changes from baseline at each evaluation time point in height velocity, height SDS, weight SDS, head circumference, body mass index (BMI), bone mineral density, BA, motor and cognitive developments measured by the BSID, IGF-I SDS, and IGFBP-3 SDS.

Safety assessments included the monitoring of adverse events and local reactions (warmth, erythema, and swelling) at the injection site obtained from the subject’s diary and laboratory tests, including metabolism and thyroid function tests.

### Statistical methods

The sample size was determined considering the rarity and prevalence of PWS. According to the data from Statistics Korea, the total births in Korea were 484,550 in 2012 [30]. The number of the pediatric patients who were newly diagnosed with PWS in Korea in 2012 was expected to be about 32 ~ 48 when the prevalence rate of PWS of 1/10,000 ~ 1/15,000 was applied [31–33]. Therefore, the number of Korean pediatric patients with PWS was expected to be less than 50 per year. Thus, the sample size was determined to be 34 (17 patients for each group) considering 15 patients for each group and a dropout rate of 10% under the practical considerations.

All subjects with treatment compliance greater than 80% during the treatment period and those who completed 52 weeks of the treatment without protocol deviations that could have a significant impact on the evaluation, were included in the efficacy analyses. Safety analyses were performed in all randomly assigned subjects who received at least 1 dose of the investigational products.

For continuous efficacy variables such as height SDS, the descriptive statistics and the two-sided 95% confidence interval (CI) for mean difference between the two groups were summarized. The percentage of subjects with at least 1 adverse event or a local reaction at the injection site was recorded, along with the number of events. For laboratory tests of safety variables, descriptive statistics were summarized, and their inter-group differences were analyzed using two sample t-test or Wilcoxon’s rank sum test. Statistical data analyses were performed using SAS® version 9.4 (SAS Institute, Inc., Cary, NC, USA).

## Results

### Subject disposition and baseline characteristics

**Fig. 1:**
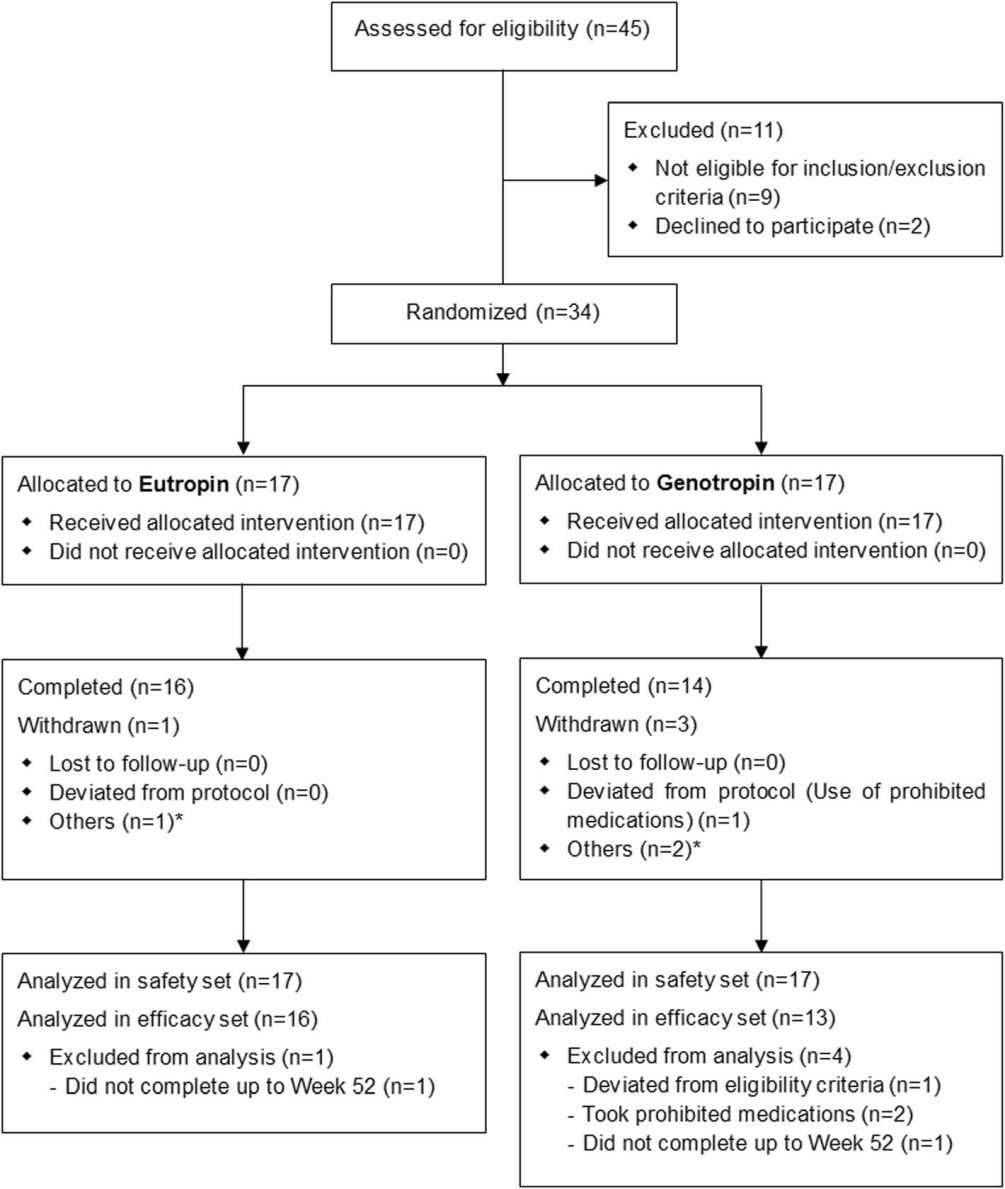
Flow chart. * The subjects (one in the Eutropin group and two in the comparator group) required the use of a prohibited medication for the treatment of adverse events, and hence, they were withdrawn from the study at the discretion of the investigator because of expected protocol deviation (use of prohibited medications)

A total of 45 patients with PWS were screened, and 34 of them were enrolled and randomly assigned to the Eutropin (*n* = 17) or the comparator (*n* = 17) group. All of them (17 in each group) received the allocated treatment as intended. Twenty-nine subjects were included in the efficacy analyses and five were excluded (1 subject who deviated from the eligibility criteria, two subjects who had used prohibited medications and two subjects who did not complete the treatment period) (Fig. 1).

Subject demographics are summarized in Table 1. Females accounted for 68.75 and 53.85% in the Eutropin and comparator groups, respectively. The mean (SD) age of participants was 4.81 (2.04) months and 8.04 (5.81) months in the Eutropin and comparator groups, respectively, with significantly younger ages in the Eutropin group than in the comparator group (*p* = 0.048). Other baseline characteristics were well balanced between the treatment groups, with the exception of height SDS, which was in average greater in the Eutropin group than in the comparator group (mean (SD) of − 1.04 (0.94) and − 2.08 (0.92), respectively, and a mean difference of 1.04 (95% CI [0.33, 1.76])), and scores for motor and cognitive development, which were higher in the comparator group than in the Eutropin group (Table 1). Mean LBM (SD) was 3438.86 (600.18) g and 3691.72 (745.93) g, and mean percent body fat (SD) was 41.53% (8.51%) and 40.04% (10.30%) in the Eutropin and comparator groups, respectively. The mean differences in LBM and percent body fat between the two groups were − 252.87 g (95% CI [− 765.33, 259.60]) and 1.49% (95% CI [− 5.67, 8.65]), respectively, with no statistical significance.

**Table 1:** Subject demographics and baseline characteristics (Efficacy set)

	Eutropin group (*N* = 16)	Comparator group (*N* = 13)	*p*-value or mean difference^a^ (95% CI)
Gender, male to female ratio	5/11	6/7	0.466^†^
Age, months			
Mean (SD)	4.81 (2.04)	8.04 (5.81)	0.048^‡^
Range	3.0–10.6	2.3–24.0	
Gestational age			0.299^†^
< 37 weeks, n (%)	1 (6.25)	3 (23.08)	
≥ 37 weeks, < 42 weeks, n (%)	15 (93.75)	10 (76.92)	
≥ 42 weeks, n (%)	0 (0.00)	0 (0.00)	
Weight at birth, kg	2.86 (0.34)	2.49 (0.48)	0.022^§^
Height SDS	−1.04 (0.94)	−2.08 (0.92)	1.04 (0.33, 1.76)
Height velocity, cm/year	21.46 (12.17)	19.51 (13.68)	1.94 (−7.91, 11.80)
Weight SDS	−1.80 (1.46)	−2.44 (1.20)	0.65 (−0.39, 1.68)
BMI, kg/m^2^	15.01 (1.92)	15.21 (2.02)	−0.20 (−1.71, 1.30)
Head circumference, cm	40.76 (1.30)	42.23 (2.45)	−1.47 (−3.06, 0.12)
LBM, g	3438.86 (600.18)	3691.72 (745.93)	−252.87 (−765.33, 259.60)
Percent body fat, %	41.53 (8.51)	40.04 (10.30)	1.49 (−5.67, 8.65)
Bone mineral density, g/cm	0.37 (0.07)	0.37 (0.07)	0.00 (−0.06, 0.05)
BA, months	3.1 (1.9)	5.6 (4.1)	−2.5 (−5.1, 0.1)
Motor development, score	14.1 (10.6)	26.2 (18.1)	−12.2 (− 23.2, − 1.1)
Cognitive development, score	28.0 (16.6)	48.5 (28.9)	−20.5 (− 39.5, − 1.6)
IGF-I SDS	−2.27 (0.07)	− 2.20 (0.19)	−0.06 (− 0.18, 0.06)
IGFBP-3 SDS	−0.89 (0.65)	− 0.71 (0.92)	−0.18 (− 0.78, 0.42)

*Abbreviations*: *CI* confidence interval, *SD* standard deviation, *SDS* standard deviation score, *BMI* body mass index, *LBM* lean body mass, *BA* bone age, *IGF-I* insulin-like growth factor I, *IGFBP-3* IGF-binding protein 3

Data are given as mean (SD) unless otherwise indicated

^a^Mean difference is Eutropin group – comparator group

^†^*p*-value obtained from Fisher’s exact test

^‡^*p*-value obtained from Wilcoxon’s rank sum test

^§^*p*-value obtained from two sample t-test

### Height SDS

**Fig. 2:**
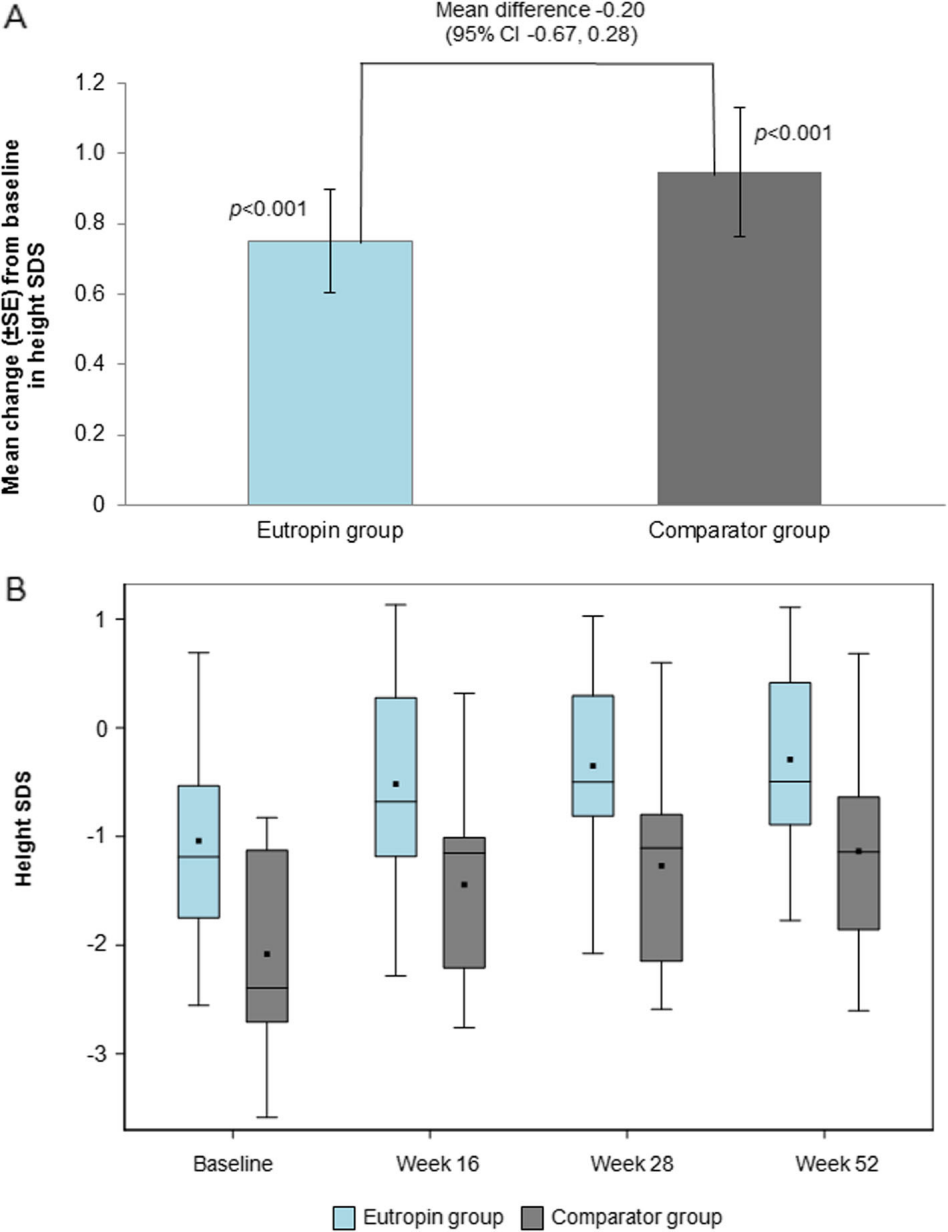
**a** Mean change in height SDS at week 52 from baseline **b** Height SDS over time. *P*-value was obtained from Paired t-test. **b** The lower and upper boundaries are the 25th percentile and the 75th percentile, respectively. The horizontal line in the box shows the median. Filled squares are mean values. SDS, standard deviation score; CI, confidence interval; SE, standard error

At week 52, the mean (SD) change in height SDS from baseline was 0.75 (0.59) and 0.95 (0.66) in the Eutropin and comparator groups, respectively, with a significant increase in both groups (*p* < 0.001). The mean difference in height SDS changes between the groups at week 52 was − 0.20 (95% CI [− 0.67, 0.28]) with no statistical significance (Fig. 2a). In addition, the analysis of covariance using the age, baseline height SDS, or weight at birth, revealed a significant difference between the two groups, as a covariate was performed, and the adjusted means of height SDS change were comparable between the two groups (Additional file 2: Table S2). The increase in height SDS over time was also similar between the two groups (Fig. 2b).

### Body composition

**Fig. 3:**
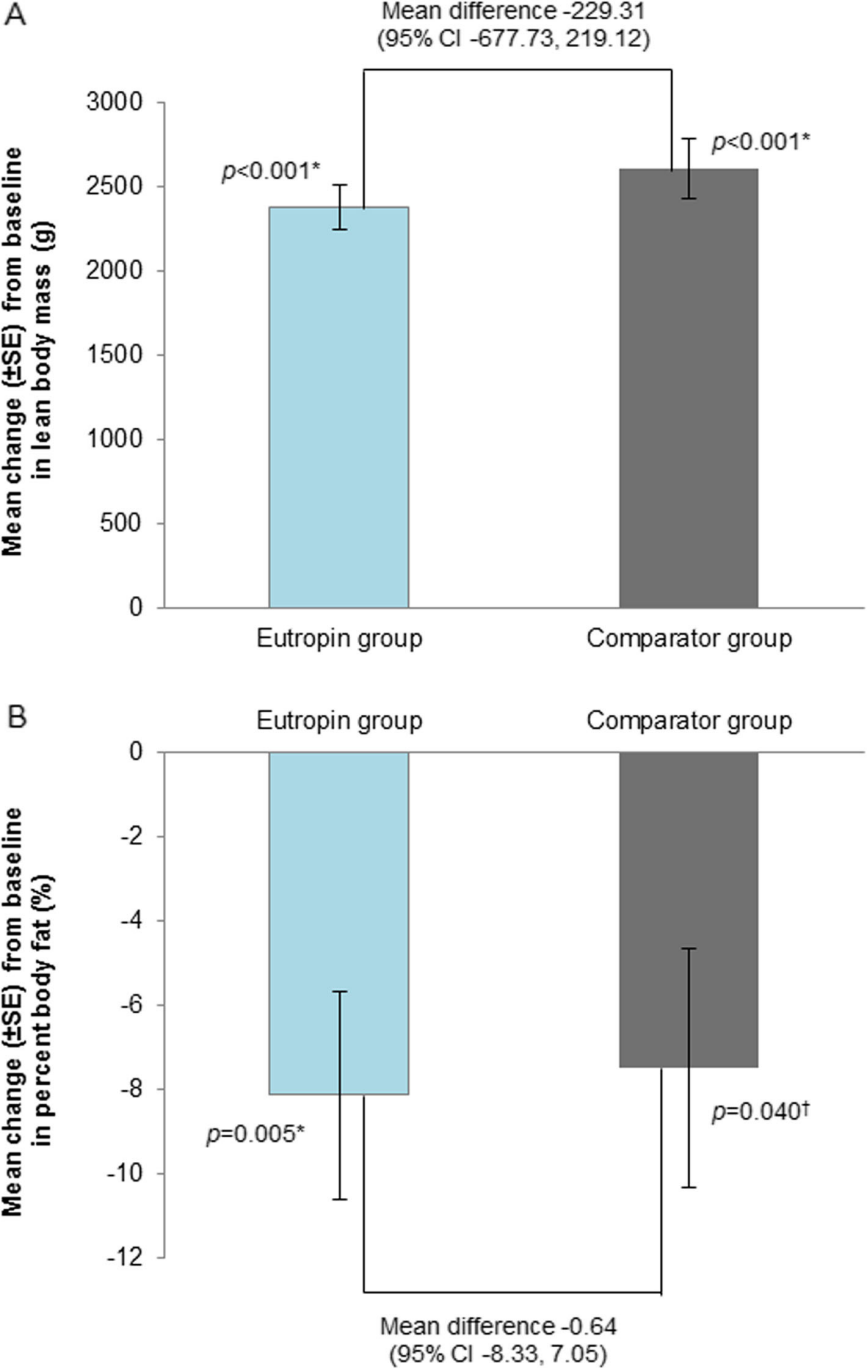
**a** Mean change in lean body mass at week 52 from baseline **b** Mean change in percent body fat at week 52 from baseline. * *P*-value was obtained from Paired t-test. ^‡^ *P*-value was obtained from Wilcoxon’s signed rank test. CI, confidence interval; SE, standard error

LBM and percent body fat results at week 52 are presented in Fig. 3. The mean (SD) changes in LBM from baseline in the Eutropin and comparator groups were 2377.79 (536.25) g and 2607.10 (641.36) g, respectively, with a significant increase at week 52 from baseline in both groups (*p* < 0.001). The mean (SD) changes in percent body fat from baseline in the Eutropin and comparator groups were − 8.12% (9.86%) and − 7.48% (10.26%), respectively, with a significant decrease at week 52 from baseline in both groups (*p* = 0.005, 0.040). The mean differences in LBM and percent body fat changes between the groups at week 52 were − 229.31 g (95% CI [− 677.73, 219.12]) and − 0.64% (95% CI [− 8.33, 7.05]), respectively, with no statistical significance. The analysis of covariance used for comparisons between the two groups adjusted for age, baseline LBM, percent body fat, or birth weight also revealed similar results (Additional file 3: Table S3 and Additional file 4: Table S4).

### Other Auxological variables

**Fig. 4:**
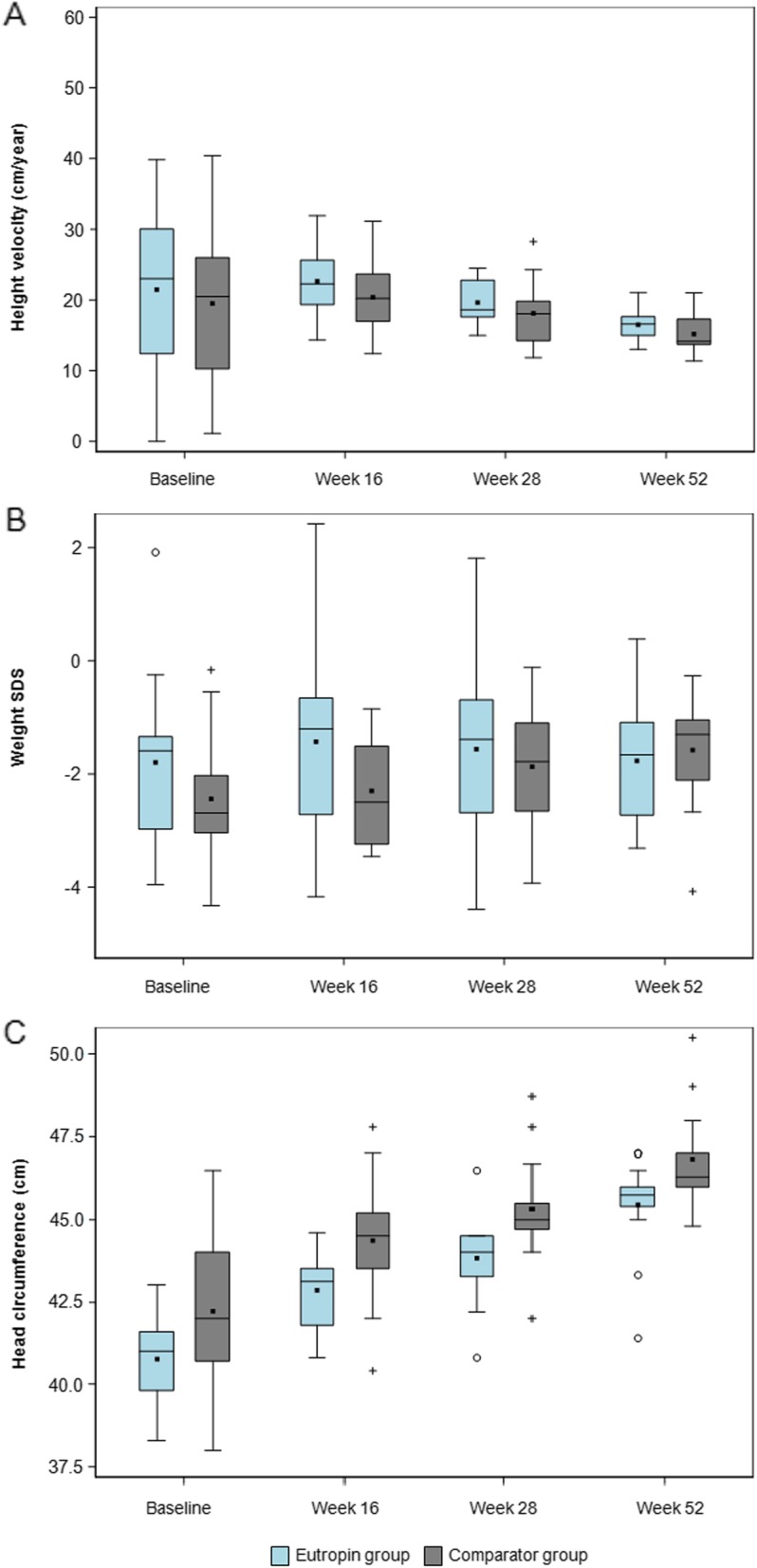
**a** Height velocity over time **b** Weight SDS over time **c** Head circumference over time. The lower and upper boundaries are the 25th percentile and 75th percentile, respectively. The horizontal line in the box shows the median. Filled squares are mean values. SDS, standard deviation score

**Fig. 5:**
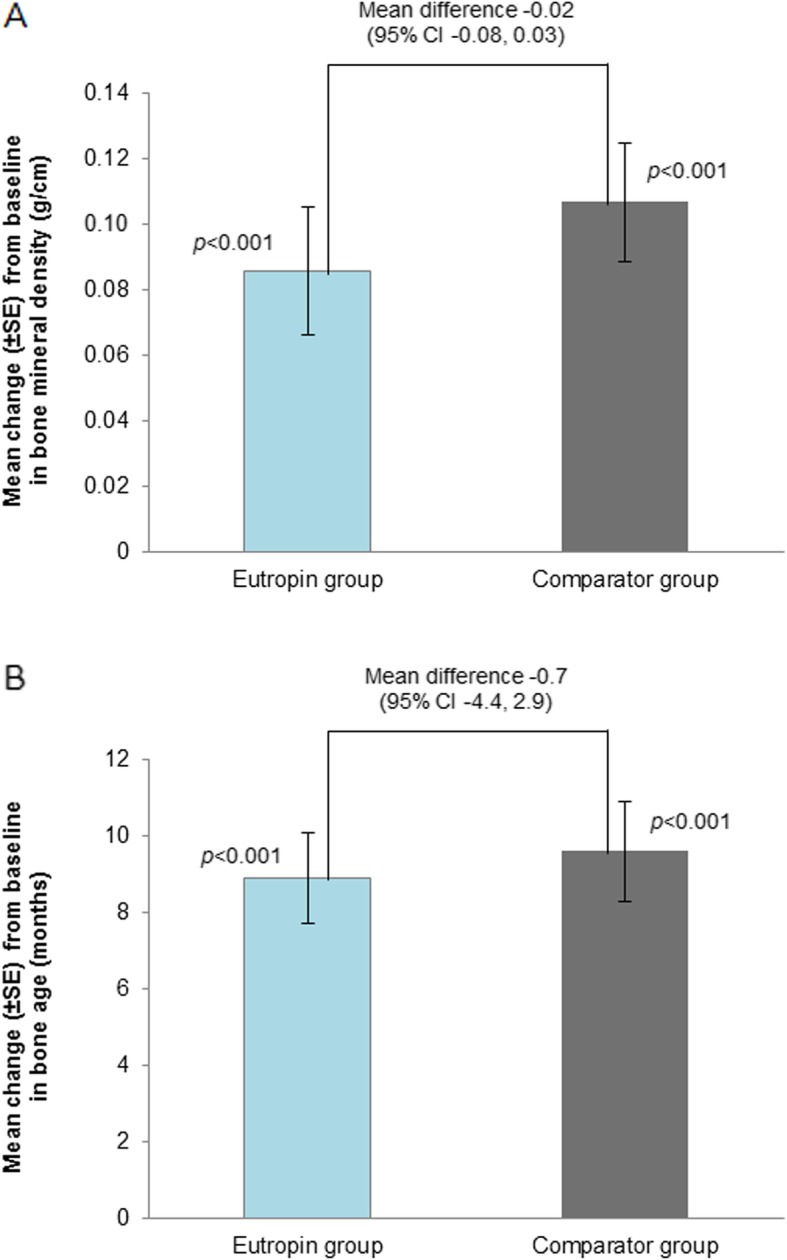
**a** Mean change in bone mineral density at week 52 from baseline **b** Mean change in bone age at week 52 from baseline. *P*-value was obtained from Paired t-test. CI, confidence interval; SE, standard error

Changes in height velocity, weight SDS, and head circumference over time were similar between the groups (Fig. 4, Additional file 5: Table S5). BMI decreased at each time point from baseline and the changes were similar between the two groups. Bone mineral density and BA were significantly increased at week 52 from baseline in both groups, and the changes were not significantly different between the groups (Fig. 5).

### Motor and cognitive developments

**Fig. 6:**
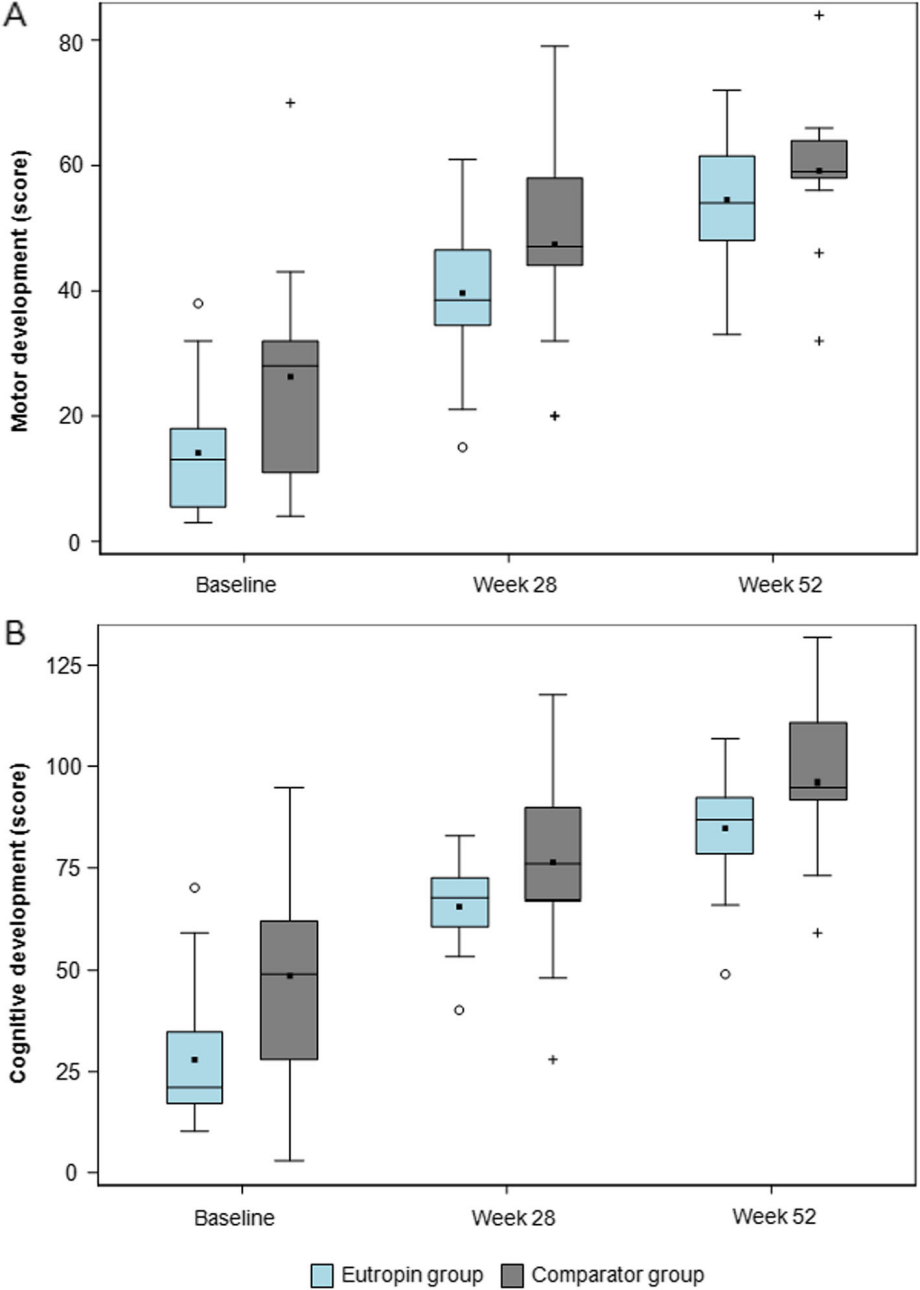
**a** Motor development score over time **b** Cognitive development score over time. The lower and upper boundaries are the 25th percentile and 75th percentile, respectively. The horizontal line in the box shows the median. Filled squares are mean values.

The mean (SD) changes in the motor development scores at week 52 from baseline were 40.4 (7.8) and 32.9 (10.5) in the Eutropin and comparator groups, respectively, and the difference between the two groups was 7.5 (95% CI [0.5, 14.5]). The mean (SD) changes in the cognitive development scores at week 52 from baseline were 56.8 (14.6) and 47.6 (13.8) in the Eutropin and comparator groups, respectively, and the difference between the two groups was 9.2 (95% CI [− 1.7, 20.1]). Both development scores indicated significant increases from baseline in both groups (*p* < 0.001), and the mean changes in motor and cognitive development scores from baseline were slightly greater in the Eutropin group than in the comparator group. Changes in motor and cognitive development scores over time are presented in Fig. 6 and the results of analysis of covariance adjusted for age on the change from baseline at week 52 are presented in Additional file 5: Table S5. The developmental percentages were also analyzed and the results are presented in Additional file 6: Table S6.

### IGF-I SDS and IGFBP-3 SDS

**Fig. 7:**
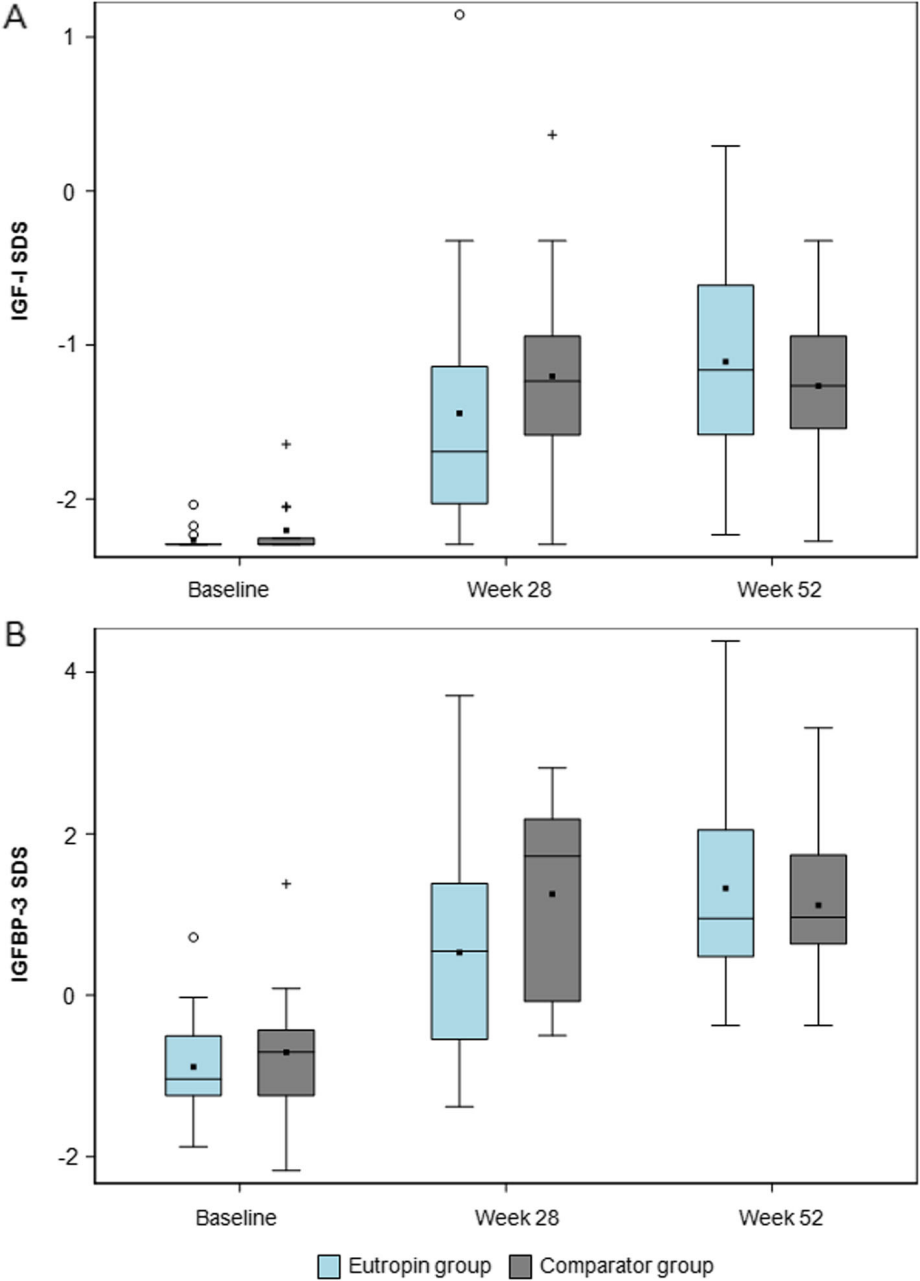
**a** IGF-I SDS over time **b** IGFBP-3 SDS over time. The lower and upper boundaries are the 25th percentile and 75th percentile, respectively. The horizontal line in the box shows the median. Filled squares are mean values. IGF-I, insulin-like growth factor I; SDS, standard deviation score; IGFBP-3, IGF-binding protein 3

Significant increases were shown in IGF-I SDS and IGFBP-3 SDS at weeks 28 and 52 from baseline in both groups, and the changes in IGF-I SDS and IGFBP-3 SDS at weeks 28 and 52 from baseline were similar between the groups (Fig. 7).

### Safety

The treatment-emergent adverse events experienced by subjects are summarized in Table 2. The incidence rate of adverse events was similar between the groups and most adverse events were mild to moderate in severity. The most frequently reported adverse event was upper respiratory tract infection, which was not related to any investigational product. A total of 15 events of adverse drug reactions were reported. Among the reported adverse drug reactions, the most frequent was hypothyroidism, with 3 and 1 events reported for the Eutropin and comparator groups, respectively, followed by two cases of decreased free thyroxine, reported in the comparator group only. Besides, 1 event of congestive cardiomyopathy was reported in the Eutropin group only; this condition was reported to be resolving at the end of the study. A total of 28 serious adverse events were reported in 15 subjects (44.12%). The incidence rate was similar between the groups, and most events were cases of pneumonia and bronchiolitis, resulting in hospitalization or extension of hospital stay. One event of sleep apnea syndrome, an adverse event of special interest, was reported in the Eutropin group only.

**Table 2:** Adverse events (Safety set)

	Eutropin group (*N* = 17)	Comparator group (*N* = 17)
Adverse events	17 (100.00)	16 (94.12)
Common adverse events (≥20% of subjects in total)		
Upper respiratory tract infection	11 (64.71)	10 (58.82)
Nasopharyngitis	7 (41.18)	7 (41.18)
Pyrexia	7 (41.18)	5 (29.41)
Pneumonia	4 (23.53)	5 (29.41)
Bronchitis	3 (17.65)	5 (29.41)
Adverse drug reactions	6 (35.29)	6 (35.29)
Common adverse drug reactions (≥5% of subjects in total)		
Hypothyroidism	3 (17.65)	1 (5.88)
Thyroxine free decreased	0 (0.00)	2 (11.76)
Serious adverse events	8 (47.06)	7 (41.18)
Infections and infestations^a^	7 (41.18)	6 (35.29)
Nervous system disorders^b^	0 (0.00)	2 (11.76)
Enteritis	1 (5.88)	1 (5.88)
Congestive cardiomyopathy	1 (5.88)	0 (0.00)
Strabismus	1 (5.88)	0 (0.00)
Middle ear effusion	0 (0.00)	1 (5.88)
Adverse events of special interest		
Sleep apnea syndrome	1 (5.88)	0 (0.00)
Upper airway obstruction	0 (0.00)	0 (0.00)
Adverse events leading to investigational product withdrawal	1 (5.88)	3 (17.65)
Local reactions at injection site	5 (29.41)	11 (64.71)
Warmth	2 (11.76)	2 (11.76)
Erythema	5 (29.41)	10 (58.82)
Swelling	2 (11.76)	2 (11.76)

Data are the number of subjects (%)

^a^Infections and infestations: bronchiolitis, bronchitis, pneumonia, upper respiratory tract infection, urinary tract infection and viral infection were included

^b^Nervous system disorders: febrile convulsion and seizure were included

A total of 4 subjects (11.76%) discontinued the treatment of the investigational product due to adverse events, namely hypothyroidism (2 subjects), decreased free thyroxine (1 subject), and seizure (1 subject). These subjects were withdrawn from the study to receive treatment for adverse events, and were reported to be recovering by the end of the study. The incidence rate of local reactions at the injection site was somewhat lower in the Eutropin group than in the comparator group. No clinically significant results were found in other laboratory tests including the metabolism test.

## Discussion

This study was conducted on very young infants and toddlers aged 2.3–24 months diagnosed with PWS. While this study included patients who had never been treated with rhGH prior to screening or who had been treated with rhGH for less than 6 months — as most of the patients in Korea are treated with rhGH shortly after they are diagnosed with PWS — it was unethical to withhold their treatment, and only young patients (less than 2 years of age) naïve to rhGH treatments were eligible for this study. In addition, for this reason, an untreated control group could not be set in the study, and this study was performed as an active-controlled design.

Height SDS and body composition (LBM and percent body fat), which are generally utilized to measure the effects of rhGH treatment in pediatric patients with PWS, were established as the primary endpoints of this study, and the changes were assessed at week 52 post rhGH administration from the baseline. It was confirmed that the administration of Eutropin to pediatric patients with PWS improves body composition and significantly increases height SDS, and it was also confirmed that Eutropin is comparable with Genotropin. In addition, the results of the secondary endpoints show trends similar to those of the primary endpoints, and in particular, motor and cognitive developments were improved.

The results of this study are in line with other studies that have examined the impact of starting GHT in infants and toddlers at a very young age [20–23, 34], supporting the premise that the early initiation of rhGH treatment in infants and toddlers with PWS provides benefits with respect to both motor and cognitive developments, that go well beyond growth and body composition. Notably, the younger the children were at the moment of GHT initiation, the greater the improvement in psychomotor development. This result suggests the importance of starting GHT before the age of 2 years, which is a critical period of child neurodevelopment (42). Moreover, recent consensus from experts recommend starting GHT soon after a diagnosis of PWS is made (as early as 3–6 months of age), as this could enhance psychomotor and cognitive functions in the long term (10, 11, 33, 36).

Although benefits of early GHT in PWS have been identified in several previous studies [34, 35], there could be some reluctance to employ GHT for very young patients with PWS. This may be owing to the broad range of challenges during infancy and owing to a lack of reports on the ideal dose and timing for treatment initiation as well as on treatment safety, for instance with regard to upper airway obstruction caused by lymphoid hypertrophy of the adenoid and/or tonsils in infants with PWS [11, 36]. However, several studies have recommended early GHT initiation, before the onset of obesity [37, 38], clinicians have not reached an agreement on the optimal age of treatment initiation. In addition, concerns have been raised with respect to the long-term effect of IGF-I levels above the reference range at the commonly recommended GH dose of 7 mg/m^2^/week or higher [11]. Our study results showed IGF-I levels adequately within the normal range, and no serious complications with standard doses of 0.24 mg/kg/week (7 mg/m^2^/week). Moreover, the changes in IGF-I SDS and IGFBP-3 SDS, which differed depending on the age of the participants, revealed a similar tendency to that found in other studies [14, 19, 21].

Overall, the incidence rate of adverse events was similar in the Eutropin and Genotropin groups, and subjects recovered from most of these events over the course of the treatment period. There was no case of upper airway obstruction (a symptom that demands special attention in the administration of rhGH) among the PWS patients who participated in this clinical trial, and sleep apnea syndrome was reported only in the Eutropin group. Since the subject who experienced sleep apnea syndrome was found to have symptoms of snoring and mouth breathing from birth, this event was evaluated to be irrelevant to the investigational product and was not considered as a serious adverse event. One event of congestive cardiomyopathy was reported in the Eutropin group; however, it occurred during the early stages of GHT and improved within 10 days, and GHT was resumed. Since then, there has been no report of any cardiac problem, and the causal relationship with the investigational product was evaluated as unlikely. However, a few previous studies have shown that long-term GHT during childhood is related to a larger aortic diameter [39], and sustained high levels of GH can lead to frank congestive heart failure [40]. In contrast, in Noonan syndrome, it is known that GHT has little impact on the progression of ventricular hypertrophy and cardiac impairment [41]. In addition, 1 reported case of dilated cardiomyopathy in adults with PWS showed a decrease in cardiac mass and function due to GH deficiency, which suggests the need for GHT in these patients [42]. However, since the clinical data are limited, regular cardiac monitoring via echocardiogram is essential during GHT.

Among the reported adverse drug reactions of hypothyroidism and decreased free thyroxine, 3 events led to discontinuation of the GHT since the prohibited concomitant medications specified in this study protocol were administered to treat the adverse events. Such abnormal results of thyroid function were shown in a previous study [24] on Genotropin. It is also notable that most of the reasons for the failure of screening in this clinical trial were central hypothyroidism with a normal thyroid stimulating hormone (TSH) value and low free thyroxine (T4) level (9 out of 11). However, even if thyroid function is normal at the time of screening, a reduction in free T4 level is likely to occur, along with a decrease in GH secretion, by the age of 2 years [43–45]. GHT itself can also increase the conversion from T4 to triiodothyronine (T3) thereby causing central hypothyroidism [46]. Therefore TSH and free T4, in addition to TSH, should be regularly monitored during GHT in pediatric patients with PWS [38].

This study has some limitations. In this study, despite the randomization, there was a slight difference in the age of the subjects between the Eutropin and Genotropin groups. However, because of the rareness of PWS, the number of eligible subjects was so small that the age of the subjects, which could influence the efficacy assessments, could not be considered as a stratification factor in the study design. Nevertheless, for the evaluation of the effects of GHT in pediatric patients with PWS in the present study, height was converted to SDS for age and sex, and the change from baseline was assessed, showing similar results in both groups. Furthermore, similar trends were observed in the analysis adjusted for age or baseline height SDS, and there was no interaction effect between the covariate (age or baseline height SDS) and treatment. In addition, age-adjusted results of the change in head circumference and motor and cognitive development were comparable between the 2 treatment groups. These results are reliable and suggest that GHT increases height SDS and improves motor and cognitive development in pediatric patients with PWS. Another limitation of this study is that nocturnal SpO_2_ monitoring was not performed to identify sleep apnea syndrome, which requires special attention during GHT. However, the patients were closely monitored for signs and symptoms related to sleep apnea syndrome by their caregivers. In addition, the small sample size and the duration of GHT (52 weeks) are also limitations of this study. Further studies would be needed to evaluate the efficacy and safety of early initiation and longer periods of GHT in a larger cohort of pediatric patients with PWS.

The strength of this study is that it was a well-organized study in which participants with PWS aged from 2.3 months to 24 months were assessed by means of DEXA, BA, and Bayley scales for infants and toddlers. Till date, there are very few reported cases of infants who have received GHT in rhGH studies. In these reports, infants have been grouped and analyzed together with toddlers [25, 47] or with 3- to 4-year-old children [48, 49]. The present study broadened the indication of GHT in PWS patients in order to provide more therapeutic options. Notably, the various ways of analysis undertaken in this study highlight the importance of the effect of GHT under the age of 2 years.

## Conclusions

Treatment with 0.24 mg/kg/week Eutropin administered subcutaneously for 52 weeks in infants and toddlers with PWS showed its efficacy in improving growth, including the increase in height SDS, body composition, and motor and cognitive development. It was also confirmed that Eutropin was generally comparable with Genotropin. In addition, the analysis on safety, including adverse events, showed no clinically significant differences between Eutropin and Genotropin. Thus, Eutropin is considered to safely improve the symptoms of pediatric patients with PWS.

## Supplementary information

**Additional file 1: Table S1.** Complete list of inclusion and exclusion criteria.

**Additional file 2: Table S2.** Analysis of covariance on the change from baseline of height SDS at week 52 (Efficacy set).

**Additional file 3: Table S3.** Analysis of covariance on the change from baseline of LBM (g) at week 52 (Efficacy set).

**Additional file 4: Table S4.** Analysis of covariance on the change from baseline of percent body fat (%) at week 52 (Efficacy set).

**Additional file 5: Table S5.** Analysis of covariance of the change from baseline to week 52 (Efficacy set).

**Additional file 6: Table S6.** Motor and cognitive developmental percentage (Efficacy set).

## Data Availability

The data supporting the findings of the study are available from the corresponding author on reasonable request.

## Abbreviations

BA: Bone age
BMI: Body mass index
BSID: Bayley scales of infant development
CI: Confidence interval
DEXA: Dual-energy x-ray absorptiometry
ECLIA: Electrochemiluminescence immunoassay
EMA: European medicines agency
GH: Growth hormone
GHT: GH treatment
IGFBP-3: IGF-binding protein 3
IGF-I: Insulin-like growth factor I
LBM: Lean body mass
PCR: Polymerase chain reaction
PWS: Prader-Willi syndrome
rhGH: Recombinant human growth hormone
SD: Standard deviation
SDS: Standard deviation score
SpO_2_: Peripheral oxygen saturation
T3: Triiodothyronine
T4: Thyroxine
TSH: Thyroid stimulating hormone
US FDA: Food and drug administration of the United States
