# Supplementary material for: Effects of recombinant human growth hormone treatment on growth, body composition, and safety in infants or toddlers with Prader-Willi syndrome: a randomized, active-controlled trial
Source: Orphanet J Rare Dis. 2019 Sep 11;14:216. doi: 10.1186/s13023-019-1195-1 (PMC6739953; doi:10.1186/s13023-019-1195-1)
Supplement: Supplementary file 1 — Additional file 1: Table S1. Complete list of inclusion and exclusion criteria. [file 13023_2019_1195_MOESM1_ESM.docx]

**Additional file 1: Table S1. Complete list of inclusion and exclusion criteria**

| Inclusion criteria |
| --- |
| 1. Pediatric patients with PWS confirmed by methylation polymerase chain reaction (PCR) genetic testing 2. Prepubertal pediatric patients (Tanner’s Pubertal stage I) at the time of screening 3. Pediatric patients who have never been treated with rhGH prior to screening, or who had been treated with rhGH for less than 6 months if they had a treatment history, and whose last administration was more than 6 months prior to screening 4. Pediatric patients with normal thyroid function at the time of screening (those with normal function through a hormonal therapy were allowed.) 5. Pediatric patients whose parents or legally authorized representatives signed the informed consent form in writing after receiving the explanation about the purpose, method, effects, etc. of the clinical study, and who also signed the informed consent form in writing if they are able to read or understand writing. |
| Exclusion criteria |
| 1. Pediatric patients with other causes of growth retardation besides PWS at the time of screening: Chronic renal failure (including cases of renal transplantation), Silver-Russell syndrome, Turner’s syndrome, Seckel syndrome, Down’s syndrome, Noonan syndrome, Cushing’s syndrome, congenital infections, psychiatric disorders, chronic debilitating diseases, etc. 2. Pediatric patients with malignancy or a history of malignancy at the time of screening 3. Pediatric patients with severe respiratory disturbance, or sleep apnoea or a history of respiratory infections with an unknown cause at the time of screening. However, those who had been confirmed to be eligible to participate in the clinical study upon the investigator’s judgment were allowed to participate in the study. 4. Pediatric patients with impaired fasting glucose, diabetes, and diabetic retinopathy at the time of screening 5. Pediatric patients whose epiphyses are closed with a growth rate of ≤1 cm/year at the time of screening 6. Pediatric patients who are being administered any drugs (estrogen, androgen, anabolic steroids, corticosteroids, GnRH analogs, thyroxine, aromatase inhibitors, etc.) that may have an effect on the secretion and actions of hGH or anticonvulsants and cyclosporin at the time of screening, or have been administered any of them for a long period of time within 6 months prior to screening (however, those who have been administered a stable dose of thyroxine preparation for 4 weeks or more [allowable in case the investigator determines the dose is stable even though it is changeable based upon the weight of the pediatric patient] were allowed to participate in the clinical study.) 7. Pediatric patients who are on any drug (e.g. methylphenidate) for the treatment of hyperactivity disorders including attention deficit hyperactivity disorder (ADHD) at the time of screening 8. Pediatric patients who are hypersensitive to somatropin or any excipient of the investigational product (cresol or glycerol) or who have a relevant history of hypersensitivity 9. Pediatric patients who have participated in any other clinical studies after enrolled in this study or who had participated in any other clinical studies within 3 months prior to enrollment in this clinical study 10. Pediatric patients in whom this clinical study is considered difficult for any other reasons on the investigator’s judgment |
