# Supplementary material for: Effects of recombinant human growth hormone treatment on growth, body composition, and safety in infants or toddlers with Prader-Willi syndrome: a randomized, active-controlled trial
Source: Orphanet J Rare Dis. 2019 Sep 11;14:216. doi: 10.1186/s13023-019-1195-1 (PMC6739953; doi:10.1186/s13023-019-1195-1)
Supplement: Supplementary file 2 — Additional file 2: Table S2. Analysis of covariance on the change from baseline of height SDS at week 52 (Efficacy set). [file 13023_2019_1195_MOESM2_ESM.docx]

**Additional file 2: Table S2. Analysis of covariance on the change from baseline of height SDS at week 52 (Efficacy set)**

|  | Eutropin group (N=16) | Comparator group (N=13) | LS mean difference* |
| --- | --- | --- | --- |
| Age (months) adjusted results |  |  |  |
| LS mean ± SE (95% CI) | 0.80 ± 0.16 (0.47, 1.13) | 0.89 ± 0.18 (0.52, 1.26) | -0.09 ± 0.25 (-0.60, 0.42) |
| Interaction test (treatment group * age), *p*-value |  |  | 0.484 |
| Baseline height SDS adjusted results |  |  |  |
| LS mean ± SE (95% CI) | 0.87 ± 0.16 (0.55, 1.19) | 0.80 ± 0.18 (0.44, 1.16) | 0.07 ± 0.25 (-0.45, 0.59) |
| Interaction test (treatment group * baseline height SDS), *p*-value |  |  | 0.978 |
| Weight (kg) at birth adjusted results |  |  |  |
| LS mean ± SE (95% CI) | 0.86 ± 0.15 (0.55, 1.17) | 0.81 ± 0.17 (0.47, 1.16) | 0.05 ± 0.24 (-0.44, 0.53) |
| Interaction test (treatment group * weight at birth), *p*-value |  |  | 0.830 |

Abbreviations: SDS, standard deviation score; LS mean, least squares mean; CI, confidence interval; SE, standard error.

* Difference is Eutropin group – comparator group.
