# Supplementary material for: Effects of recombinant human growth hormone treatment on growth, body composition, and safety in infants or toddlers with Prader-Willi syndrome: a randomized, active-controlled trial
Source: Orphanet J Rare Dis. 2019 Sep 11;14:216. doi: 10.1186/s13023-019-1195-1 (PMC6739953; doi:10.1186/s13023-019-1195-1)
Supplement: Supplementary file 3 — Additional file 3: Table S3. Analysis of covariance on the change from baseline of LBM (g) at week 52 (Efficacy set). [file 13023_2019_1195_MOESM3_ESM.docx]

**Additional file 3: Table S3. Analysis of covariance on the change from baseline of LBM (g) at week 52 (Efficacy set)**

|  | Eutropin group (N=16) | Comparator group (N=13) | LS mean difference* |
| --- | --- | --- | --- |
| Age (months) adjusted results |  |  |  |
| LS mean ± SE (95% CI) | 2355.60 ± 153.45 (2040.18, 2671.03) | 2634.41 ± 171.55 (2281.78, 2987.04) | -278.81 ± 238.45 (-768.96, 211.34) |
| Interaction test (treatment group * age), *p*-value |  |  | 0.889 |
| Baseline LBM (g) adjusted results |  |  |  |
| LS mean ± SE (95% CI) | 2366.52 ± 149.40 (2059.42, 2673.62) | 2620.98 ± 166.07 (2279.62, 2962.33) | -254.46 ± 225.43 (-717.83, 208.92) |
| Interaction test (treatment group * baseline LBM), *p*-value |  |  | 0.825 |
| Weight (kg) at birth adjusted results |  |  |  |
| LS mean ± SE (95% CI) | 2437.21 ± 151.33 (2126.16, 2748.27) | 2533.97 ± 169.60 (2185.34, 2882.60) | -96.76 ± 238.13 (-586.24, 392.73) |
| Interaction test (treatment group * weight at birth), *p*-value |  |  | 0.657 |

Abbreviations: LBM, lean body mass; LS mean, least squares mean; CI, confidence interval; SE, standard error.

* Difference is Eutropin group – comparator group.
