# Supplementary material for: Effects of recombinant human growth hormone treatment on growth, body composition, and safety in infants or toddlers with Prader-Willi syndrome: a randomized, active-controlled trial
Source: Orphanet J Rare Dis. 2019 Sep 11;14:216. doi: 10.1186/s13023-019-1195-1 (PMC6739953; doi:10.1186/s13023-019-1195-1)
Supplement: Supplementary file 4 — Additional file 4: Table S4. Analysis of covariance on the change from baseline of percent body fat (%) at week 52 (Efficacy set). [file 13023_2019_1195_MOESM4_ESM.docx]

**Additional file 4: Table S4. Analysis of covariance on the change from baseline of percent body fat (%) at week 52 (Efficacy set)**

|  | Eutropin group (N=16) | Comparator group (N=13) | LS mean difference* |
| --- | --- | --- | --- |
| Age (months) adjusted results |  |  |  |
| LS mean ± SE (95% CI) | -8.20 ± 2.65 (-13.64, -2.76) | -7.38 ± 2.96 (-13.46, -1.30) | -0.82 ± 4.11 (-9.28, 7.63) |
| Interaction test (treatment group * age), *p*-value |  |  | 0.602 |
| Baseline percent body fat (%) adjusted results |  |  |  |
| LS mean ± SE (95% CI) | -7.51 ± 1.36 (-10.31, -4.71) | -8.23 ± 1.51 (-11.34, -5.12) | 0.72 ± 2.04 (-3.48, 4.91) |
| Interaction test (treatment group * baseline percent body fat), *p*-value |  |  | 0.568 |
| Weight (kg) at birth adjusted results |  |  |  |
| LS mean ± SE (95% CI) | -8.33 ± 2.68 (-13.83, -2.82) | -7.22 ± 3.00 (-13.39, -1.06) | -1.10 ± 4.21 (-9.76, 7.55) |
| Interaction test (treatment group * weight at birth), *p*-value |  |  | 0.260 |

Abbreviations: LS mean, least squares mean; CI, confidence interval; SE, standard error.

* Difference is Eutropin group – comparator group.
