# Supplementary material for: Effects of recombinant human growth hormone treatment on growth, body composition, and safety in infants or toddlers with Prader-Willi syndrome: a randomized, active-controlled trial
Source: Orphanet J Rare Dis. 2019 Sep 11;14:216. doi: 10.1186/s13023-019-1195-1 (PMC6739953; doi:10.1186/s13023-019-1195-1)
Supplement: Supplementary file 5 — Additional file 5: Table S5. Analysis of covariance of the change from baseline to week 52 (Efficacy set). [file 13023_2019_1195_MOESM5_ESM.docx]

**Additional file 5: Table S5. Analysis of covariance of the change from baseline to week 52 (Efficacy set)**

|  | Eutropin group (N=16) | Comparator group (N=13) | LS mean difference* (95% CI) |
| --- | --- | --- | --- |
| Age (months) adjusted results on the change from baseline at week 52 |  |  |  |
| Head circumference, cm |  |  |  |
| LS mean change ± SE | 4.5 ± 0.3 | 4.9 ± 0.3 | -0.4 (-1.3, 0.6) |
| Motor development, score |  |  |  |
| LS mean change ± SE | 38.6 ± 1.9 | 35.2 ± 2.1 | 3.3 (-2.7, 9.4) |
| Cognitive development, score |  |  |  |
| LS mean change ± SE | 54.3 ± 3.2 | 50.7 ± 3.6 | 3.6 (-6.6, 13.8) |

Abbreviations: LS mean, least squares mean; CI, confidence interval; SE, standard error.

* Difference is Eutropin group – comparator group.
