# Supplementary material for: Effects of recombinant human growth hormone treatment on growth, body composition, and safety in infants or toddlers with Prader-Willi syndrome: a randomized, active-controlled trial
Source: Orphanet J Rare Dis. 2019 Sep 11;14:216. doi: 10.1186/s13023-019-1195-1 (PMC6739953; doi:10.1186/s13023-019-1195-1)
Supplement: Supplementary file 6 — Additional file 6: Table S6. Motor and cognitive developmental percentage (Efficacy set). [file 13023_2019_1195_MOESM6_ESM.docx]

**Additional file 6: Table S6. Motor and cognitive developmental percentage (Efficacy set)**

|  | Eutropin group (N=16) | Comparator group (N=13) | Mean difference* (95% CI) |
| --- | --- | --- | --- |
| Motor development, %^†^ |  |  |  |
| Baseline | 38.8 (11.9) | 43.7 (16.0) | -4.9 (-15.6, 5.7) |
| Week 52 | 53.5 (14.4) | 51.1 (11.4) | 2.3 (-7.7, 12.4) |
| Percentage of change^‡^ | 45.3 (44.1) | 34.7 (76.0) | 10.6 (-39.4, 60.5) |
| Cognitive development, %^†^ |  |  |  |
| Baseline | 44.3 (18.8) | 56.4 (19.6) | -12.1 (-26.8, 2.5) |
| Week 52 | 64.9 (15.7) | 67.4 (14.2) | -2.5 (-14.0, 9.0) |
| Percentage of change^‡^ | 66.4 (71.3) | 29.1 (39.4) | 37.4 (-5.8, 80.5) |

Abbreviations: CI, confidence interval.

Data are given as mean (SD) unless otherwise indicated.

* Difference is Eutropin group – comparator group.

^†^ Motor and cognitive development % = {(developmental age/chronological age) × 100}.

^‡^ Percentage of change = [{(development at Week 52–development at baseline)/development at baseline} × 100].
